# Positron Emission Tomography in Prostate Cancer: Summary of Systematic Reviews and Meta-Analyses

**DOI:** 10.18383/j.tom.2015.00130

**Published:** 2015-09

**Authors:** Hossein Jadvar

**Affiliations:** Division of Nuclear Medicine, Department of Radiology, Keck School of Medicine, University of Southern California, Los Angeles, CA

**Keywords:** prostate, cancer, positron emission tomography, meta-analysis

## Abstract

Prostate cancer is a prevalent public health problem worldwide. Over the past decade, there has been tremendous research activity in the potential use of positron emission tomography with a number of radiotracers targeted to various biological aspects of this complex tumor. Systematic reviews and meta-analyses are important contributions to the relevant literature that summarize the evidence while reducing the effect of various sources of bias in the published data. The accumulation of relevant data in this clinical setting has recently provided the opportunity for systematic reviews. In this brief article, I summarize the published systematic reviews and meta-analyses of positron emission tomography in prostate cancer. Most robust evidence suggests a probable role for first-line use of positron emission tomography with radiolabeled choline in restating patients with biochemical relapse of prostate cancer with the diagnostic performance that seems to be positively associated with the serum prostate-specific antigen level and velocity. Future systematic reviews will be needed for other emerging radiotracers such as those based on the prostate-specific membrane antigen and gastrin-releasing peptide receptor.

## Introduction

Several studies have investigated the potential utility of positron emission tomography (PET) with different radiotracers in prostate cancer. These radiotracers are based on the underlying complex biology of the tumor with several agents that have been evaluated in men in various phases of the disease (e.g., initial diagnosis, staging, restaging during biochemical recurrence, treatment response assessment, prognosis, and outcome prediction). These include 18F-fluorodeoxyglucose (FDG; glucose metabolism), 11C-acetate and 18F- or 11C-choline (lipogenesis), anti-1-amino-3-[18F]fluorocyclobutane-1-carboxylic acid (anti-18F-FACBC; amino acid metabolism), 16a-18F-fluoro-5a-dihydrotestosterone (targeted to androgen receptor), and more recently radiotracers based on gastrin-releasing peptide receptor and prostate-specific membrane antigen, among others targets (1).

Although there have been many narrative review articles on this evolving topic, formalized systematic reviews and meta-analyses have only recently appeared in the literature for a few radiotracers (Table 1). Systematic reviews and meta-analyses involve formulating a detailed search strategy and evaluating and statistically combining relevant evidence to produce a quantitative estimate of various performance parameters of interest (e.g., pooled sensitivity, specificity, etc.) (2). The goal of this brief article is to bring together a summary of the recently published systematic reviews and meta-analyses of PET in the imaging evaluation of prostate cancer.

**Table 1.** PET Radiotracers in Prostate Cancer With Published Systematic Review and Meta-Analysis

PET Radiotracer	Biological Basis for Uptake	Phase of Disease	Reference
18F-fluorodeoxyglucose	Glucose metabolism	Incidental prostatic uptake	4
11C-acetate	Lipogenesis in cellular membrane biosynthesis	Primary tumor; biochemical recurrence	7
18F- and 11C-choline	Lipogenesis in cellular membrane biosynthesis	Initial staging; biochemical recurrence (and effect of PSA level and kinetics)	11-15
Anti-1-amino-3-[18F]fluorocyclobutane-1-carboxylic acid	Amino acid metabolism	Biochemical recurrence	18

Abbreviations: PET, positron emission tomography; PSA, prostate-specific antigen.

## PET Imaging in Prostate Cancer

### Imaging Glucose Metabolism with FDG

**Figure 1.**
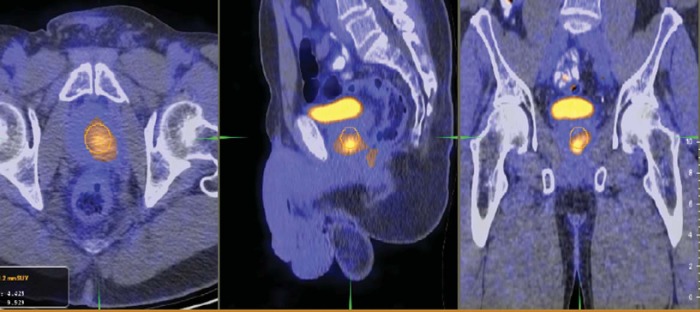
Axial, sagittal, and coronal fused positron emission/computed tomography image (left-to-right panels) shows incidental high 18F-fluorodeoxyglucose uptake (maximum standard uptake value of 7.7) in the right prostate lobe of a 67-year-old man who presented for restaging colon cancer; subsequent workup revealed a serum prostrate-specific antigen level of 14.6 ng/mL and a biopsy-proven prostate cancer with a Gleason score of 8.

FDG is the most common radiotracer in PET scintigraphy. The biological basis for increased FDG uptake in malignancy is related to the Warburg effect, one of the hallmarks of cancer (3). The only published systematic review and meta-analysis with FDG as the radiotracer in the clinical setting of prostate cancer is related to the prevalence and risk of malignancy of prostatic incidental uptake of FDG (4). In 6 studies that included 47 925 patients, the pooled prevalence of incidental prostatic FDG uptake was 1.8% (95% confidence interval [CI]: 1.3%–2.3%). The pooled risk of malignancy in patients with incidental prostatic FDG uptake verified by biopsy was 62% (95% CI: 54%–71%). The authors concluded that incidental peripheral prostatic FDG uptake might carry a significant probability of malignancy (Figure 1).

### Imaging Lipogenesis with Radiolabeled Acetate and Choline

PET with radiolabeled acetate and choline can image lipogenesis in prostate cancer. Acetate participates in the de novo synthesis of fatty acids from acetyl-CoA and malonyl-CoA through the upregulated action of fatty acid synthase in support of increased malignancy-related cell membrane biosynthesis (5, 6). In a systematic review and meta-analysis of 11C-acetate PET/computed tomography (CT) in prostate cancer, 23 studies were included (7). For evaluating the primary tumor, pooled sensitivity and specificity were 75.1% (95% CI: 69.8%–79.8%) and 75.8% (95% CI: 72.4%–78.9%), respectively. For detecting recurrence, pooled sensitivity was 64% (59%–69%), and pooled specificity was 93% (95% CI: 83%–98%). Moreover, sensitivity for detecting recurrence was higher in postsurgical versus postradiotherapy patients and in patients with serum prostate-specific antigen (PSA) at a relapse of >1 ng/mL.

Most prostrate cancer studies have employed radiolabeled choline (8). Choline is an essential nutrient that was discovered by Adolph Strecker in 1862 (9). He noted that upon heating of lecithin from bile, a gelatinous substance is formed that he called choline. Lecithin was later characterized chemically as phosphatidylcholine, which was then found to incorporate choline (10). Dietary choline is absorbed in the intestine by choline transporters and then phosphorylated to phosphocholine (catalyzed by choline kinase) or oxidized to betaine in some cells. Two other enzymes in sequence further catalyze phosphocholine to form phosphatidylcholine, which is then incorporated into the cellular membranes. Given that tumors grow rapidly and proliferate, there is a demand for increased cellular membrane synthesis, which forms the basis for the observed increased radiolabeled choline uptake in some tumors. Choline may also accumulate in some benign conditions. As such, choline uptake in tissues is not cancer-specific.

Choline may be labeled with 11C (half-life: 20 minutes) or 18F (half-life: 110 minutes). The physiologic biodistribution of 11C-choline and 18F-fluorocholine are similar (relatively high uptake in liver, pancreas, kidney, salivary glands, and variable uptake in intestine) except that there is low renal tracer excretion with 11C-choline; hence, there is less bladder urine activity accumulation. For both tracers, the critical organ receiving the highest dose is the kidney. The US Food and Drug Administration has approved only 11C-choline for local production and use at the Mayo Clinic in Rochester, Minnesota, for restaging prostate cancer (11).

Umbehr et al. provided a systematic review and meta-analysis of 11C-choline and 18F-fluorocholine PET for the initial staging of prostate cancer. They reported a pooled sensitivity and specificity on a per patient basis (10 studies, 637 patients) of 84% (95% CI: 68%–93%) and 79% (95% CI: 53%–93%), respectively (12). In restaging patients with biochemical recurrence, they reported a pooled sensitivity and specificity on a per patient basis (12 studies, 1 055 patients) of 85% (95% CI: 79%–89%) and 88% (95% CI: 73%–95%), respectively (7). A similar report by von Eyben et al. examined 47 articles and data from 3 167 patients with regard to the diagnostic utility of choline PET/CT in staging and restaging of prostate cancer (13). They found that there were statistically significant more positive results in the prostate bed of biochemically relapsed patients who had previously undergone external beam radiation therapy rather than those patients who had radical prostatectomy as the initial treatment strategy. Moreover, choline PET/CT led to a change in treatment in 381 (41%) of 938 patients, leading to complete PSA response in 101 of 404 (25%) patients.

**Figure 2.**
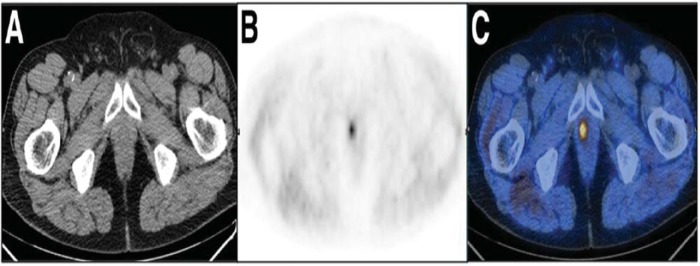
A 68-year-old man with previously diagnosed prostate cancer (Gleason score of 8) who was treated with definitive radical prostatectomy and presented with biochemical relapse (PSA, 0.85 ng/mL; PSAdt, 7.4 m; PSAvel, 0.94 ng/mL/y). 11C-choline PET/CT (A, CT; B, PET; C, PET/CT) revealed locally recurrent disease in the surgical bed. CT, computed tomography; PET, positron emission tomography; PSA, prostrate-specific antigen; PSAdt, PSA doubling time; PSAvel, PSA velocity. [Reproduced with permission from Castellucci et al (21).]

Another systematic review and meta-analysis by Evangelista et al. (1,555 patients across 19 studies, including 12 for all sites of disease, 3 for lymph node metastases, and 4 for local recurrence) on the use of choline PET and PET/CT in the biochemical relapse of prostate cancer reported a pooled sensitivity of 85.6% (95% CI: 82.9%–88.1%) and specificity of 92.6% (95% CI: 90.1%–94.6%) for all sites of disease (prostatic fossa, lymph nodes, and bone); a pooled sensitivity of 75.4% (95% CI: 66.9%–82.6%) and specificity of 82% (95% CI: 68.6%–91.4%) for prostatic fossa recurrence; and a pooled sensitivity of 100% (95% CI: 90.5%–100%) and specificity of 81.8% (95% CI: 48.2%–97.7%) for lymph node metastases (14). The reported 100% pooled sensitivity for detecting lymph node metastases may have been overestimated given the small number of publications that were included in the meta-analysis (Figure 2).

It has been noted that the diagnostic performance of choline PET/CT may depend on serum PSA level and kinetics. Treglia et al. performed a systematic review of 14 articles specifically focused on the relationship between PSA level and kinetics (e.g., PSA doubling time [PSAdt] and PSA velocity [PSAvel]) on the lesion detection rate in restaging prostate cancer (15). The overall pooled detection rate of choline PET/CT in restaging prostate cancer was 58% (95% CI: 55%–60%). The pooled detection rate increased to 65% (95% CI: 58%–71%) when PSAdt was ≤6 months and to 71% (95% CI: 66%–76%) and 77% (95% CI: 71%–82%) when PSAvel was >1 or >2 ng/mL/y, respectively. Shen et al. reported the results of a meta-analysis (16 articles consisting of 27 studies) that compared the diagnostic performance of choline PET/CT, magnetic resonance imaging (MRI), bone single-photon emission-computed tomography, and planar bone scintigraphy (BS) in detecting bone metastases in parents with prostate cancer (16). On a per patient basis, the pooled sensitivities for choline PET/CT, MRI, and BS were 91% (95% CI: 83%–96%), 97% (95% CI: 91%–99%), and 79% (95% CI: 73%–83%), respectively. The pooled specificities for detecting bone metastases using choline PET/CT, MRI, and BS were 99% (95% CI: 93%–100%), 95% (95% CI: 90%–97%), and 82% (95% CI: 78%–85%), respectively.

### Imaging Amino Acid Metabolism with Anti-18F-FACBC

Anti-18F-FACBC is a synthetic nonmetabolized analogue of the amino acid leucine that accumulates in prostate cancer cells through the upregulated alanine, serine, cysteine (ASC) transport system (17). More recently, it has also been observed that androgens augment the expression of the ASC transporter system, thereby increasing the uptake of anti-18F-FACBC in prostate cancer (18).

**Figure 3.**
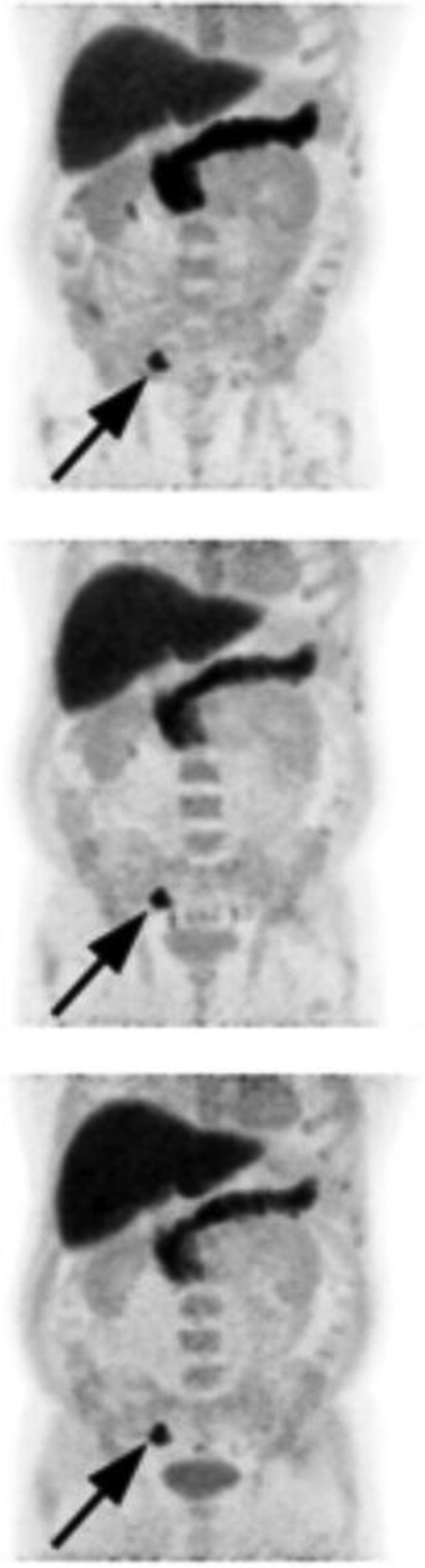
Maximum-intensity-projection anti-1-amino-3-[18F]fluorocyclobutane-1-carboxylic acid images acquired over intervals of 5 to 16 minutes (top), 17 to 28 minutes (middle), and 29 to 40 minutes (bottom) in a man with metastatic recurrent prostate cancer in right iliac nodes (arrow). Note physiologic intense tracer uptake in liver and pancreas and increasing bladder activity over time. [Reproduced with permission from Schuster et al (22)]

Ren et al. reported on a systematic review and meta-analysis of 6 studies comprising 251 patients who were suspected of having recurring prostate carcinoma and who underwent anti-18F-FACBC PET/CT. The authors reported pooled sensitivity and specificity of 87% and 66% on a per patient-based analysis, respectively, in detecting prostate carcinoma recurrence (19) (Figure 3).

### Comparison of PET Radiotracers in Prostate Cancer

The relatively large array of potential PET radiotracers and paucity of comparative studies in specific clinical situations in prostate cancer lead to both challenges and opportunities. It may be that a particular radiotracer is best suited for a particular phase of the disease while another may be most appropriate for another phase along the natural clinical history of prostate cancer. Our group at the University of Southern California recently reported on a comprehensive extracting and reanalysis of the PET detection data for FDG, 11C-acetate, 11C- or 18F-choline, and anti-18F-FACBC (20). We found that FDG exhibited the lowest detection rate for any suspected disease. In addition, 11C-acetate tended to be advantageous over radiolabeled choline in detecting local recurrence and lymph node lesions, although the difference was not statistically significant. Anti-18F-FACBC had the greater likelihood of detecting local recurrence compared with radiolabeled choline, although again this difference was not statistically significant.

## Summary

Overall, the most robust evidence is for the probable first-line use of choline PET/CT in restating prostate cancer patients who have biochemically relapsed. More evidence and experience will be needed for the other candidate PET radiotracers (e.g., those targeted to prostate-specific membrane antigens, the gastrin-releasing peptide receptor, and others) in the imaging evaluation of prostate cancer. Other pertinent issues such as availability, access, regulatory, and reimbursement hurdles will also need to be resolved.

**Disclosure:** The author declares no conflicts of interest. Parts of this article appeared in the Society of Nuclear Medicine and Molecular Imaging PET Center of Excellence Newsletter Vol.12, Issue 1, 2015.
